# Recent advances in lateral flow devices and point-of-care diagnostics for highly pathogenic avian influenza A viruses

**DOI:** 10.1128/jvi.01484-25

**Published:** 2025-11-04

**Authors:** Meng-Wei Lin, Irwin A. Quintela, Shyam S. Sablani, Chih-Sheng Lin, Vivian C. H. Wu

**Affiliations:** 1 Produce Safety and Microbiology Research Unit, United States Department of Agriculture, Agricultural Research Service 230648, Albany, California, USA; 2 Biological Systems Engineering, Washington State University 6760, Pullman, Washington, USA; 3 Department of Biological Science and Technology, National Yang Ming Chiao Tung University 34914, Hsinchu, Taiwan; 4 Center for Intelligent Drug Systems and Smart Bio-devices (IDS2B), National Yang Ming Chiao Tung University 34914, Hsinchu, Taiwan; Indiana University Bloomington, Bloomington, Indiana, USA

**Keywords:** HPAI, avian influenza, H5N1, lateral flow assay, CRISPR

## Abstract

There has been a resurgence and ongoing outbreak of avian influenza since early 2024. Avian influenza, caused by influenza A viruses, poses significant threats to both avian populations and public health due to its zoonotic potential. Highly pathogenic avian influenza (HPAI) virus, such as the H5N1 and H7 subtypes, has a high mortality rate. Traditional detection methods, i.e., virus isolation and reverse transcription quantitative PCR (qRT-PCR), are reliable for diagnosis but time-consuming and labor-intensive. Rapid and accurate detection of avian influenza A viruses is crucial to prevent widespread outbreaks and minimize economic losses. Field-ready and point-of-care (POC) diagnostics, such as lateral flow assays (LFAs) offer a rapid, early, and large-scale approach for detecting avian influenza A virus infections. Early detection in HPAI management is a key factor in improving treatment effectiveness and reducing the negative impact on animal health. This minireview introduces the principles and techniques of current field-ready and POC diagnostics, emphasizing LFAs for HPAI detection. It also outlines and compares their development, applications, and availability in the market. Notably, advanced techniques such as LFAs-integrated clustered regularly interspaced short palindromic repeats (CRISPR) have expanded HPAI diagnostic capabilities and have also been reviewed. CRISPR-based LFAs use guide RNA to detect viral sequences, activating Cas enzymes that generate a visible signal on test strips, enabling a rapid and sensitive detection method. To our knowledge, this is the first comprehensive review summarizing LFA-based HPAI diagnostics in the context of the 2024 resurgence, offering timely insight into their potential roles in outbreak preparedness and response.

## INTRODUCTION

Avian influenza, commonly known as bird flu, is an infectious viral disease affecting bird species worldwide (1, 2). Caused by influenza A viruses, avian influenza poses significant threats to both avian populations and public health due to its potential for zoonotic transmission (3). Particularly, highly pathogenic avian influenza (HPAI), subtypes like H5N1 and H7 viruses, exhibit a high mortality rate (4). The rapid and accurate detection of avian influenza viruses (AIVs) is crucial for implementing control measures, preventing widespread outbreaks, and minimizing economic losses in the poultry industry (5, 6).

H5N1 was first detected in geese in Guangdong, China, 1996 (7). The first human H5N1 cases were described in Hong Kong in 1997 (8). In recent years, the global landscape of avian influenza has been marked by a series of notable outbreaks (Table 1). In May 2021, the HPAI H5N1 virus was detected in wild foxes at a rehabilitation center in the Netherlands during an outbreak of HPAI in wild birds (9). From late 2021 to 2022, the predominant HPAI H5 virus causing poultry outbreaks worldwide was the wild bird-adapted HPAI H5N1 virus, according to the World Organization for Animal Health (10). In 2022, HPAI H5N1 re-emerged in commercial poultry, with a U.S. outbreak in turkeys and two asymptomatic human cases reported in Spain among poultry workers exposed to infected flocks, marking the first U.S. detection since 2020 and raising concern over zoonotic transmission (10, 11).

**TABLE 1:** Timeline of avian influenza H5N1 outbreaks & key events

Year	Avian influenza H5N1 outbreaks & key events
1996–1997	First H5N1 alarm
1996	H5N1 first detected in geese in Guangdong, China
1997	Hong Kong outbreak: first documented human infections with H5N1 (18 infected, 6 deaths); authorities cull ~1.5 million poultry
2003–2004	Re-emergence and global spread
2003	H5N1 re-emerges in Asia (Hong Kong, Vietnam, Thailand, South Korea)
2004	Massive poultry outbreaks and human cases across Southeast Asia; over 100 million birds culled
2005–2006	Spread to Europe, Middle East, Africa
2005	Qinghai Lake, China: major wild bird die-off; virus spreads via migratory routes
2006	H5N1 detected in Europe (e.g., Germany, France), Africa (Nigeria, Egypt), and the Middle East
2020–2023	Unprecedented Global H5N1 outbreak
2020	New strain of H5N1 appears in Europe and spreads rapidly
2021–2023	Millions of birds culled worldwide Wild bird populations (e.g., seabirds, raptors were) heavily affected First confirmed mammal infections (e.g., minks in Spain, sea lions in South America) Limited human cases (e.g., U.S., UK, Cambodia), but no sustained human-to-human
2022	Second documented human infection with H5N1 in Spain
2023	Two human cases of H5N1 in Cambodia
2024	H5N1 outbreak in dairy cattle
	First-ever detection of HPAI (H5N1) in dairy cattle in Texas, U.S; the FDA confirms that the H5N1 virus was detected in retail milk samples, though pasteurization inactivates the virus; no risk to consumers was reported
2025	Ongoing surveillance
	Over 35 countries have reported avian influenza outbreaks H5N1 clade 2.3.4.4b remains the dominant strain worldwide Mammal-to-mammal transmission potential is increasing

Between 2023 and early 2025, H5N1 continued to expand its host range and geographic impact. In February 2023, Cambodia reported two human infections, including the death of a child, linked to clade 2.3.2.1c (12). By December, H5N1 was identified in a polar bear in the Arctic (10). In March 2024, the U.S. detected H5N1 in dairy cows, followed by human infections in dairy and poultry workers (13). From September to December 2024, North America reported 45 human cases of clade 2.3.4.4b (14). As of February 2025, over 34 countries have reported outbreaks, disrupting poultry industries, while Brazil’s exports surged amid global shortages (15).

Early detection is essential for effective disease management, enabling timely control measures and reducing the impact on animal health. Recent events highlight the evolving nature of highly pathogenic avian influenza viruses (HPAIVs) and reinforce the urgent need for robust surveillance systems and reliable diagnostic tools. Traditional diagnostic methods for HPAIVs, such as virus isolation and reverse transcription-PCR (RT-PCR), are highly sensitive and specific (16, 17). However, they require specialized laboratory facilities and trained personnel, which are time-consuming and may delay outbreak response efforts (18). In contrast, lateral flow assays (LFAs) offer a rapid, user-friendly, and cost-effective alternative for the on-site detection of HPAIVs (19, 20). LFAs are designed to detect specific antigens or antibodies in samples, providing results within minutes without requiring complex equipment (21).

The application of LFAs in avian influenza detection has gained momentum, especially in resource-limited settings and during field investigations. Their portability and ease of use make them invaluable for immediate decision-making and implementation of control measures. Moreover, advancements in LFA technology have led to improved sensitivity and specificity, enhancing its reliability as a diagnostic tool.

This review provided a comprehensive overview of the application of LFAs in avian influenza detection, focusing on HPAI strains. In light of the resurgence and ongoing outbreaks since early 2024, impacting poultry, dairy cattle, and even leading to zoonotic infections, there is an urgent need for rapid, accessible diagnostics that complement traditional laboratory-based methods such as virus isolation and reverse transcription quantitative PCR (qRT-PCR). LFAs offer practical advantages for early detection, large-scale screening, and on-site decision-making during outbreaks. To our knowledge, this is the first comprehensive review of LFA-based diagnostics for HPAI conducted in the context of the 2024 outbreak. As HPAI continues to threaten both animal and human health, integrating rapid diagnostic tools like LFAs into surveillance programs will be pivotal in mitigating the impact of future outbreaks.

## ZOONOTIC HIGHLY PATHOGENIC AVIAN INFLUENZA A VIRUSES AND THE TROPISM

**Fig 1:**
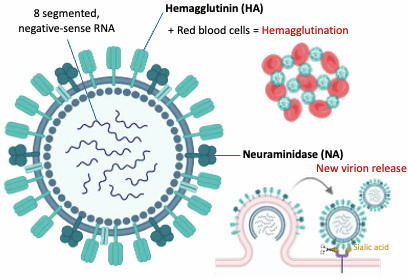
Illustration of the structure of AIV and the functional interactions between its envelope proteins and host cells. Hemagglutination occurs when the AIV’s HA binds to sialic acid receptors on red blood cells (RBCs), causing the RBCs to clump together in a lattice-like network. Conversely, NA facilitates viral release by removing sialic acids from both host cell receptors and newly formed HA and NA on emerging viral particles. This figure was created with BioRender (https://www.biorender.com/).

Influenza A viruses belong to the Orthomyxoviridae family and possess an eight-segmented, negative-sense RNA genome (22). The virus comprises eight gene segments encoding essential proteins, including hemagglutinin (HA) and neuraminidase (NA), which determine viral subtype and host specificity (23) (Fig. 1). The segmented genome allows reassortment, facilitating the emergence of novel strains with zoonotic and pandemic potential (24).

**Fig 2:**
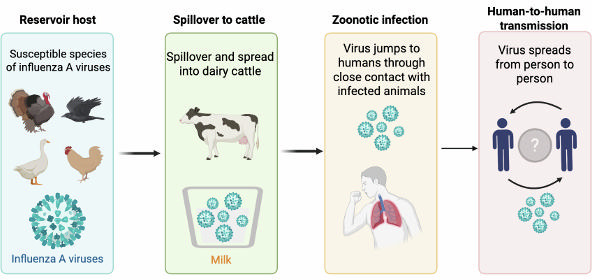
Illustration of the spillover and spread of AIVs into dairy cattle and humans. A reassortment event in a poultry host resulted in the emergence of the AIVs. After spilling over into dairy cattle, the virus successfully established infection, enabling efficient cow-to-cow transmission (intraspecies transmission) and transmission to other species, including humans (interspecies transmission). However, the potential for human-to-human transmission remains uncertain. This figure was created with BioRender (https://www.biorender.com/).

Influenza A viruses are divided into subtypes based on two surface proteins of the virus – HA and NA. There are 18 known HA subtypes and 11 known NA subtypes (25, 26). Various subtypes of AIVs have been associated with zoonotic transmission, causing sporadic human infections and posing pandemic threats (27). These viruses primarily circulate among birds but have demonstrated the ability to infect humans, sometimes leading to severe disease outbreaks (28). Continuous surveillance and research are essential to understand their evolution, transmission, and potential impact on public health (Fig. 2).

### H5N1 viruses

HPAI H5N1 has caused severe outbreaks in poultry and sporadic human cases since its emergence in 1997 in Hong Kong (29). This virus has demonstrated a high fatality rate in humans, often exceeding 50%, making it a significant concern for public health (30). Transmission occurs primarily through direct contact with infected birds or contaminated environments, with no sustained human-to-human transmission reported to date (31). However, the virus continues to evolve through genetic reassortment and mutation, raising concerns over its pandemic potential (32).

H5N1 has spread across multiple continents, affecting poultry farms, wild birds, and, occasionally, mammals (33). Several clades and subclades of H5N1 have been identified, each with varying levels of virulence and transmissibility (34, 35). H5N1 primarily infects the respiratory and gastrointestinal tracts of birds, causing severe systemic infections (30). In humans, H5N1 preferentially binds to α2,3-linked sialic acid receptors found in the lower respiratory tract, leading to severe pneumonia, acute respiratory distress syndrome (ARDS), and multiorgan failure (36). The virus has been isolated from the brain, intestines, and other organs, indicating neurotropism and systemic dissemination (37). Its ability to infect mammals, including humans, raises concerns about zoonotic spillover and pandemic potential (33, 38) (Table 2).

**TABLE 2:** Common zoonotic avian influenza A viruses and their host tropism

Subtype	Species	Organs	Publishing year	Reference
H5N1	Poultry	Multiple internal organs	2007	(39)
	Bovine	Pulmonary and mammary tissues	2024	(40)
	Human	Respiratory and gastrointestinal tracts	2024	(36)
H1N1	Swine	Intestines	2013	(41)
	Human	Upper respiratory tract	2025	(42)
H7N9	Poultry	Respiratory and gastrointestinal tracts	2013, 2015	(43, 44)
	Human	Lower respiratory tract	2013, 2018	(45, 46)
H9N2	Poultry	Respiratory and gastrointestinal tracts	2025	(47)
	Human	Upper respiratory tract	2018	(48)

Efforts to control H5N1 include poultry vaccination programs, culling infected flocks, and strict biosecurity measures. Moreover, antiviral treatments such as oseltamivir have been used in infected individuals, though resistance remains a concern. Given its ongoing evolution, continuous surveillance and research are critical to mitigating the risks associated with H5N1.

### H7N9 viruses

H7N9 is an avian influenza virus that emerged in humans in China in 2013, causing severe respiratory illness with a high mortality rate (49). H7N9 was classified as a low pathogenic avian influenza virus (LPAIV) in birds, meaning infected poultry may show little to no symptoms (50). However, in March 2017, the United States Department of Agriculture (USDA) confirmed the presence of HPAI H7N9 in a broiler chicken breeder flock in the USA (51). Besides, when transmitted to humans, the virus can cause severe pneumonia, ARDS, and multi-organ failure (49). The case fatality rate of H7N9 has varied across outbreaks, with some waves of infection exhibiting mortality rates above 30% (52).

H7N9 predominantly infects the respiratory and gastrointestinal tracts of avian species (43, 44). In humans, H7N9 exhibits a preference for α2,3-linked sialic acid receptors, primarily found in the lower respiratory tract, contributing to severe pneumonia and ARDS (45, 46). The virus has shown evidence of limited systemic spread, with detection in blood and other organs. Its ability to bind both avian and human receptors suggests a risk for adaptation and potential human-to-human transmission (53, 54) (Table 2).

Genetic studies have revealed that H7N9 possesses mutations that enhance its binding affinity to human respiratory receptors, increasing concerns about its pandemic potential (55). Vaccination efforts have been implemented in poultry to reduce the virus’s circulation, and antiviral treatments, including NA inhibitors, remain the primary therapeutic options for infected individuals (56). Continuous monitoring and research are essential to prevent further zoonotic spillovers and potential adaptation for human-to-human transmission.

### Other subtypes

H1N1 is also known for zoonotic and reassortment potential, infects humans and animals (42). The 2009 pandemic highlighted its adaptability, stressing the need for vaccination, biosecurity, and ongoing surveillance to prevent outbreaks (57). H1N1 primarily targets the respiratory epithelium, binding to both α2,3- and α2,6-linked sialic acid receptors, facilitating interspecies transmission (58). In swine, H1N1 replicates efficiently in the intestines, enabling fecal-oral transmission (41). In humans, it infects the upper respiratory tract, leading to mild to severe respiratory illness (42).

H9N2 is a widespread LPAIV that circulates among poultry worldwide (59). In poultry, H9N2 primarily replicates in the respiratory and gastrointestinal tracts, causing mild to moderate disease (47). Human infections are typically mild, with virus replication observed in the upper respiratory tract due to its affinity for both α2,3- and α2,6-linked sialic acid receptors (48). Although it primarily affects birds, sporadic human infections of H9N2 have been reported, particularly in individuals with occupational exposure to infected poultry (60). H9N2 infections in humans typically result in mild respiratory symptoms, but the significance lies in the role as a genetic donor to other zoonotic influenza viruses (61). H9N2 viruses have been implicated in the evolution of highly pathogenic strains, such as H5N1 and H7N9, through genetic reassortment (62, 63). The virus possesses internal gene segments that have contributed to the emergence of novel influenza strains with increased human infectivity (64).

While H5, H7, and H9 avian influenza A viruses are most commonly associated with human infections (65), other subtypes pose zoonotic risks. Historical examples include the 1957 H2N2 Asian flu, the 1968 H3N2 Hong Kong flu, and the 1977 Russian flu (66). More recently, an avian-origin H4N6 virus was detected in US pigs in 2015 (67), and H5N6 infections have been reported in children (68). The ability of these viruses to reassort highlights the need for ongoing surveillance. Furthermore, some H10N4 and H10N8 viruses have demonstrated the capacity to bind to human-like receptors (69). H11N9 viruses can be transmitted directly to hunters from ducks (70). These avian influenza subtypes primarily circulate in birds, but human infections occur through exposure to infected birds or contaminated environments (71). Most AVIs exhibit a strong affinity for α2,3-linked receptors, limiting human-to-human transmission but posing a zoonotic threat (58, 72). The diverse host tropism of these subtypes underscores the ongoing risk of interspecies transmission and potential pandemics.

Therefore, global surveillance, robust biosecurity measures, and effective vaccination strategies are crucial for minimizing zoonotic risks. A deeper understanding of the genetic and epidemiological characteristics of these viruses is essential for predicting and preparing for future outbreaks.

## FIELD-READY AND POINT-OF-CARE (POC) TESTING FOR HPAI

The progression of avian influenza from low pathogenic to highly pathogenic strains in poultry was first recorded in domestic geese in 1996 (73, 74), and the emergence of HPAI viruses of the H5N1 subtype in various animal species presents a potential pandemic risk (75). While qRT-PCR coupled with spin column RNA extraction remains the gold standard for HPAI virus surveillance (76), other influenza virus detection and diagnostic testing methods such as (1) rapid tests for antigen, (2) rapid molecular assays for viral RNA, (3) immunofluorescence assays, (4) viral culture, and (5) serological tests can also be implemented. As the demand for on-site assays grows, point-of-care (POC) testing has become a viable alternative to traditional methods, mainly due to its fast results, ease of use, affordability, and minimal infrastructure requirements. One of the main types of POC testing devices is paper-based technology. Paper-based devices are more suitable for simpler, often single-step reactions and are less expensive, which makes them widely used in areas with limited resources. Among the different paper-based POC options – dipsticks, LFA, and microfluidic devices, LFA stands out due to its ease of production, simple operation, robustness, and user-friendliness. Lateral flow assay utilizes capillary action coupled with binding or capture elements to detect the target analyte(s) in a strip format. Due to its demonstrated advantages, LFA has garnered significant interest from researchers, inventors, and healthcare professionals, expanding its applications across various POC fields.

Recent research underscores the potential of the clustered regularly interspaced short palindromic repeats (CRISPR) system, the CRISPR sequences and associated nucleases system, as an advanced platform for POC testing (6). These systems have been applied to detect a wide range of targets, including viruses, bacteria, parasites, cancer mutations, genotypes, and small molecules, positioning CRISPR technology at the forefront of next-generation diagnostics (77, 78). In particular, immune-based methods and CRISPR-Cas13a-based detection technologies have garnered significant attention for HPAI (79). CRISPR-based approaches have been successfully integrated into LFA systems at the laboratory scale, demonstrating strong potential for early-stage detection of HPAI during outbreak scenarios.

### Antibody-based LFAs for HPAI

Immuno-based serological tests, such as enzyme-linked immunosorbent assay (ELISA) can detect elicited antibodies, which are a direct result of the host’s immune response to infection. Immunoglobulin G (IgG) ELISAs have been used to measure the recognition and binding of antibodies to a wide range of seasonal and avian HA proteins (80). However, these tests may not be reliable, especially during the early stages of infection, due to their detection limit. On the other hand, antigen detections of HA and NA proteins of HPAI viruses via ELISA can be utilized at the initial phase of an outbreak but require specialized equipment, reagents, and technical expertise (81, 82).

Rapid and precise diagnosis is one of the key approaches to control HPAI viruses, but the sensitivity of HPAI diagnostic tools has progressively reduced over time due to broad antigenic variations during the evolution of HPAI viruses. To overcome this, Nguyen, Nakaishi (83) designed a rapid LFA detection kit by combining two anti-H5 HA monoclonal antibodies (mAbs), (1) A64/from Linjudge Flu A/H5 and (2) the novel mAb A32/2, which was generated from clade 2.3.4.4 H5 HPAI. This new approach has improved the sensitivity and specificity of the original Linjudge Flu A/H5, as demonstrated by its successful detection of antigens from swabs and tissue homogenates of naturally and experimentally infected birds with H5N6 HPAIVs from the genetic clade 2.3.4.4 (10^2.2^–10^3.4^ TCID_50_/test). No cross-reactivity was observed when a panel of 18 IAV reference strains (H1–H16 subtypes) and two strains of influenza B viruses were tested. Recently, Mata Calidonio, Maddox (75) designed a low-cost paper-based immunoassay that can detect H5N1 HA protein from a more comprehensive set of HPAI-related sample matrices such as sera from humans, sheep, horses, poultry, dairy products (eggs and milk), and wild birds (oral, cloacal, and fecal samples). This study also included a strain belonging to HPAI H5 clade 2.3.4, which still possesses similar antigenic properties to the descendant clade 2.3.4.4 and accounts for most cases of the current outbreak. Functionalized gold nanospheres with α-HA IgG Abs established a direct colorimetric response with a limit of detection (LOD) of 0.16 nM and 1.72 nM in human serum and whole milk, respectively. However, the immunoassay did not perform relatively well when a highly viscous matrix (heavy cream) was tested (Table 3).

**TABLE 3:** Published LFAs used to detect HPAI recently^*a*^

Reference	Subtype	Real sample	LFA type	LOD
(83)	H5 clade 2.3.4.4	10-day-old embryonated chicken egg	Antibody	TCID_50_: 10^2.2^–10^3.4^
(75)	H5 clade 2.3.4	Milk; chicken egg; human, horse, and sheep serum; oral, cloacal, fecal swab of wild birds	Antibody	0.16 nM (human serum);1.72 nM (milk)
(84)	H9N2; H5N9; H1N1	Chicken egg; chicken	Antibody	EID_50_: 2.9 × 10^6^ (egg);10^4.865^ (chicken)
(79)	H5	Poultry virus nucleic acid samples	CRISPR-Cas13a	0.1 copy/μL
(85)	H5; H7; H9	Clinical samples	CRISPR-Cas13a	1 copy/μL
(86)	H5; H7; H9	Throat swab samples from diseased chickens	CRISPR-Cas13a	10^1^ copies/µL
(87)	H5; H7; H9	Throat swab samples from diseased chickens	RT-RAA	10^1^ copies/µL
(82)	H5Nx	Fecal samples collected from migratory bird	CRISPR-Cas12a	EID_50_: 10^1^ (fluorescence);EID_50_: 10^2^ (lateral flow)

^*a*^ LFA: lateral flow assay; LOD: limit of detection; TCID_50_: 50% tissue culture infectious dose; EID_50_: 50% egg infective dose; RT-RAA: reverse transcription recombinase-aided amplification.

The viral internal nucleoprotein (NP) of HPAI has also become a target for diagnostic development since it is highly conserved among AVIs with less susceptibility to mutations as compared to HA and NA proteins (84, 88, 89). A porous silica nanoparticle-based chemiluminescent LFA system with signal-amplifiable capability was designed by Lee, Kim (84) to detect NP of HPAI (H5N9). This LFA system was able to detect 20- to 100-fold lower AIV levels from chickens’ cloacal and oropharyngeal swab samples than a commercial rapid kit/method with 10^4^ (50% egg infective dose; EID_50_)/mL LOD. Its signal-amplifiable sensing probes rely on the optimum pore sizes of silica nanoparticles, such as sizable cavities, where larger biomolecules, such as antibodies, were conjugated onto the outer wall, increasing the chance of binding with HPAI’s NPs. However, since NP is concentrated inside the envelope of HPAI viruses, an additional lysis step is needed to facilitate its release (Table 3).

 There are commercially available immune-based LFA systems specifically designed for qualitatively detecting H5N1 antigens, such as Avian Influenza Virus H5N1 Antigen Lateral Flow Assay Kit (Elabscience, Houston, TX, USA) for tracheal or cloacal poultry secretions. Another gold immunochromatographic assay, Avian Influenza H5N1 Virus Rapid Test Kit (Abbexa, Cambridge, UK), can also qualitatively detect H5N1 antigens from the stool and saliva of chickens. Cows that are infected by H5N1 often display clinical symptoms such as respiratory distress, decreased appetite, altered stool consistency, reduced and anomalous milk production (90). Milk samples can be tested for H5N1 antigen using the AIV-H5N 1 Antigen Rapid Test Kit (Reagen, San Diego, CA, USA) in conjunction with auxiliary diagnosis of infection. Furthermore, the USDA approved one commercial product FluDETECT Avian (Zoetis, Parsippany, NJ, USA), for chicken and turkey. These conventional commercial immune-based LFA systems generate line signals when the target antigens are more abundant than the LOD. However, non-specific binding can be caused by sample matrix components such as proteins and salts that may adhere to the nitrocellulose membrane or Abs. Additionally, fecal and tissue lysates may clog the membranes and create uneven flow, leading to artifact formation (Table 4).

**TABLE 4:** Summary of commercial LFA kits for HPAI^*a*^

Company	Country	Product	Reactivity	Sample type	LFA type	Official approval
Elabscience	USA	Avian Influenza Virus Antigen Lateral Flow Assay Kit	Poultry	Saliva; feces	Antibody	NA
Abbexa	UK	Avian Influenza H5 Virus Antigen Rapid Test Kit	Chicken	Saliva; feces	Antibody	NA
Reagent	USA	AIV-H5N1 Antigen Rapid Test Kit	Cow	Milk	Antibody	NA
MEDIAN	Korea	DRG AIV Ag Rapid kit 2.0	Chicken; duck	Feces; oropharyngeal swab tissue homogenate	Antibody	NA
VET Diagnostix	China	Avian Influenza Virus Antigen Test	Bird	Saliva; feces	NA	NA
HWTAi	China	Avian Influenza Virus H5 Serotype Antigen Rapid Test	Avian	Serum; secretions; spleen	Antibody	NA
Zoetis	USA	FluDETECT Avian	Chicken; turkey	Saliva; feces	Antibody	USDA

^*a*^ NA, not applicable.

### CRISPR-based LFA for avian influenza

The CRISPR system is a powerful gene-editing technology derived from a bacterial immune defense mechanism that uses RNA-guided enzymes, Cas proteins, to precisely recognize and cut specific DNA or RNA sequences (91). When integrated into LFAs, CRISPR-based platforms use guide RNA to identify viral or bacterial sequences, activating Cas enzymes that produce a detectable signal on the test strip (92). This combination allows rapid, sensitive, and portable detection of pathogens at the point of care, enhancing traditional LFA capabilities with molecular-level precision. Detecting DNA and RNA from various environmental sources (e.g., water, feces, etc.) is a promising approach to monitor potential wildlife pathogens with limited disturbance to the organisms or their habitats. Minimally invasive methods like swabbing or brushing animals and noninvasive methods such as environmental DNA sampling show great potential for surveying various species, including hidden invasive and at-risk species (93, 94). CRISPR-based diagnostics are powerful tools that can successfully detect unique DNA and RNA sequences from associated target pathogens; however, their applications in wildlife disease management have been sluggish (94). LFA has been investigated as a compatible platform for integrating CRISPR-Cas13a technology into field-ready/POC diagnostics and transforming its results into visible and easy-to-interpret signals.

Li et al. (79) designed a recombinase-aided amplification (RAA) coupled with CRISPR-Cas13a and LFA system for detecting H5 avian influenza virus from clinical samples. The methods achieved a LOD of 0.1 copy/μL, which was in line with qRT-PCR. No cross-reactivity with other AVIs (H3, H7, H9, and H10), was observed. Similarly, Yang, Yang (85) developed two rapid detection methods for avian influenza virus based on CRISPR-Cas13a. These methods detect avian influenza virus through the M gene and identify H5, H7, and H9 subtypes via the HA gene. The first uses reverse transcription (RT)-RAA with Cas13a for amplification and qRT-PCR, achieving a LOD of 1 copy/μL. The second technology combines reverse transcription recombinase-aided amplification (RT-RAA) with Cas13a and a LFA system, achieving a LOD of 10 copies/μL. Both methods show high sensitivity and specificity, potentially surpassing qRT-PCR for clinical use. A very similar approach was implemented by Zhang, Wang (86), which tested additional non-targets such as infectious bronchitis virus, infectious laryngotracheitis virus, and Newcastle disease virus, wherein no cross-reactivity was reported. Moreover, clinical samples (e.g*.,* throat swabs) from individual chickens presenting mild or severe respiratory symptoms associated with avian influenza infection were also tested for avian influenza virus H5, H7, and H9 using RT-RAA-LF dipstick. They showed a LOD of 10^1^ copies/µL – consistent with the results of real-time fluorescence qRT-PCR (87), but this approach was employed, excluding the CRISPR-Cas13a method (Table 3).

For the centrifuge-free H5Nx avian influenza detection method, Song et al. (76) combined magnetic bead-based RNP purification using anti-NP antibodies with a PAM-independent CRISPR-Cas12a system, enabled by DMSO-mediated amplicon denaturation. This eliminates spin columns and specific PAM sequences, increasing flexibility. The assay detected H5Nx with high specificity, achieving sensitivities of 10^1^ EID_50_ (fluorescence) and 10^2^ EID_50_ (lateral flow), and clinical sample sensitivity of 80% and specificity of 100%. This approach offers a promising on-site diagnostic tool for rapidly mutating RNA viruses (Table 3).

Pre-amplification method (e.g., RAA) for nucleic acids increases the overall sensitivity of CRISPR-Cas even in the presence of PCR interferents. By combining CRISPR’s ability to precisely target specific nucleic acids with the user-friendliness and speed of LFA, the resulting platform offers accurate, fast, cost-effective, and portable testing for HPAI and a wide range of disease-causing viral agents. Their combination excels in speed, sensitivity, and accessibility and is highly adaptable for both field-ready and point-of-care diagnostics.

While CRISPR-based LFAs for HPAI are not yet commercially available on the market, active research investigations are currently exploring this technology, and it may only be a matter of time before these highly sensitive and rapid tests are deployed for detecting HPAI.

## CHALLENGES AND LIMITATIONS OF LFA-BASED TECHNOLOGY FOR AVIAN INFLUENZA DETECTION

LFA-based technology has been widely adopted for virus detection due to its rapid results, ease of use, and cost-effectiveness (19). However, several challenges and limitations hinder its broader application in disease surveillance and outbreak control. A primary concern with conventional antibody-based LFAs is their suboptimal sensitivity and specificity, especially when detecting low-abundance analytes or distinguishing closely related viral targets (95). These limitations are particularly problematic in early-stage infections, where low viral loads can result in false negatives, and cross-reactivity with other influenza subtypes may lead to false positives (96, 97). Furthermore, environmental factors such as temperature and humidity may impact test performance, reducing reproducibility in field conditions (98).

Another challenge is the quantitative limitation of traditional LFAs. Most LFA-based tests provide qualitative or semi-quantitative results, making it difficult to assess viral load, which is crucial for determining infection severity and guiding disease management strategies (99). Additionally, current LFA technologies often struggle to differentiate between highly pathogenic and low pathogenic avian influenza strains, which is vital for disease control measures (100). Determination between highly pathogenic and low pathogenic avian influenza A virus still relies on nucleotide sequencing or mass spectrometry (101, 102).

To address these challenges, recent advances in POCT have introduced promising innovations (103). CRISPR-based LFAs, in particular, offer improved sensitivity, specificity, and adaptability, helping to overcome the shortcomings of traditional assays (104). Future POCT developments may incorporate nanomaterials, smartphone-based analysis, and CRISPR-Cas technology to enhance sensitivity, specificity, and quantitative capabilities. Integration with internet of Things and artificial intelligence-driven diagnostics can also improve real-time data analysis and epidemiological tracking (105–107).

While LFA-based technology remains a valuable tool for avian influenza detection, addressing its current limitations through advanced POCT innovations will enhance its effectiveness in disease surveillance and outbreak management. Future research should focus on improving detection accuracy, quantification capabilities, and real-time connectivity to strengthen global avian influenza control efforts.

In the regulatory context, point-of-care pathogen diagnostics used in veterinary settings are classified as veterinary biologics and fall under the jurisdiction of the USDA’s Center for Veterinary Biologics (108). These products must meet specific sensitivity and specificity criteria to be approved for distribution. However, reference laboratory assays, including those for biomarkers, hormones, complete blood counts, and clinical chemistry panels, are currently not regulated by the USDA (109). Furthermore, there appears to be no standardized regulatory framework for animal disease detection tools, highlighting the need for clearer guidelines as diagnostic technologies evolve.

## CONCLUSION

LFAs have emerged as valuable tools for the rapid detection of avian influenza, providing a cost-effective and user-friendly alternative to conventional diagnostic methods. However, their broader utility in disease surveillance is currently limited by sensitivity, quantification, and strain differentiation challenges. Emerging innovations, such as CRISPR-based detection systems, smartphone-integrated platforms, and AI-enabled data analysis, hold promise for overcoming these limitations. Ongoing research and technological advancements continue to enhance the performance of LFAs, improving their reliability for field applications. Future efforts should focus on increasing sensitivity, expanding multiplexing capabilities, and integrating LFAs with digital platforms for real-time data sharing and surveillance.
